# Racial, ethnic, and sex disparities in buprenorphine treatment from emergency departments by discharge diagnosis

**DOI:** 10.1111/acem.70035

**Published:** 2025-04-25

**Authors:** Neeraj Chhabra, Dale Smith, Natalie Parde, Nicole Hsing-Smith, Joseph M. Bianco, R. Andrew Taylor, Gail D'Onofrio, Niranjan S. Karnik

**Affiliations:** 1 Department of Emergency Medicine, University of Illinois Chicago, Chicago, Illinois, USA; 2 AI.Health4All Center for Health Equity Using Machine Learning and Artificial Intelligence, Chicago, Illinois, USA; 3 Institute for Research on Addictions, University of Illinois Chicago, Chicago, Illinois, USA; 4 Department of Psychiatry, University of Illinois Chicago, Chicago, Illinois, USA; 5 Natural Language Processing Laboratory, Department of Computer Science, University of Illinois Chicago, Chicago, Illinois, USA; 6 University of Illinois College of Medicine, Chicago, Illinois, USA; 7 Department of Emergency Medicine, Yale University School of Medicine, New Haven, Connecticut, USA; 8 Department of Medicine, Yale School of Medicine, New Haven, Connecticut, USA; 9 Department of Epidemiology (Chronic Disease), Yale School of Public Health, New Haven, Connecticut, USA

## Abstract

**Objectives:**

Racial and sex disparities are noted in the administration and prescribing of buprenorphine from emergency departments (EDs) nationally. It is unknown whether disparities persist when accounting for the specific discharge diagnosis addressed during encounters such as opioid overdose or withdrawal.

**Methods:**

We conducted a cross-sectional analysis of opioid-related ED encounters from January 2020 through December 2023 using a national database, Epic Cosmos. We analyzed the effect of opioid encounter subtype—overdose or withdrawal—on receipt of buprenorphine using multivariable logistic regression adjusting for demographics and measured confounding variables. Encounter subtypes were defined by diagnosis codes and buprenorphine receipt was defined as administration or prescribing. We evaluated for racial, ethnic, and sex disparities within encounter subtypes for withdrawal and overdose.

**Results:**

We examined 1,088,033 opioid-related encounters. Adjusted odds for buprenorphine receipt were greater for encounters involving withdrawal (odds ratio [OR] 2.22, 95% CI 2.18–2.26), though reduced for overdose (OR 0.52, 95% CI 0.51–0.53) and other opioid complications (OR 0.69, 95% CI 0.64–0.70). Males were more likely to receive buprenorphine (OR 1.18, 95% CI 1.16–1.19) than females. All racial minorities excepting American Indian/Native American patients (OR 1.04, 95% CI 1.00–1.08) were less likely to receive buprenorphine than White patients (Asian OR 0.85, 95% CI 0.79–0.81; Black OR 0.80, 95% CI 0.79–0.81; Native Hawaiian/Pacific Islander OR 0.79, 95% CI 0.71–0.89). Subtype analyses indicated decreased odds for buprenorphine receipt for female patients across all subtypes. An increased odds for buprenorphine receipt among Black patients (OR 1.04, 95% CI 1.01–1.07; ref. White race) was noted in encounters involving opioid withdrawal but disparities persisted for opioid overdose.

**Conclusions:**

The administration and prescribing of buprenorphine in the ED is heavily influenced by the presence of opioid withdrawal. Disparities disadvantage female patients and racial minorities. Some racial disparities, particularly among Black patients, are not evident when solely considering encounters involving opioid withdrawal. System-level interventions are needed to address disparities and improve the equitable uptake of ED-initiated buprenorphine.

## INTRODUCTION

Drug overdose is the leading cause of accidental death in the United States, with the majority attributable to opioids.^1^ Medications for opioid use disorder (MOUD), such as buprenorphine, decrease morbidity and mortality in patients with OUD.^2,3^ However, only one in four Americans with opioid use disorder (OUD) receive MOUD, and nearly half of Americans with OUD receive no treatment at all.^4^

Patients with substance use disorders disproportionately utilize emergency services.^5,6^ The low-barrier accessibility of emergency departments (EDs) make them critical access points for patients with OUD to initiate buprenorphine treatment.^7^ However, individual ED practices vary, and disparities exist in buprenorphine administration and prescribing from the ED.^8-10^

In the United States, female patients with OUD are less likely to receive buprenorphine than male patients in the ED.^9,10^ Additionally, Black patients and other racial minorities are less likely to be administered or receive a prescription for buprenorphine than White patients in the ED setting.^9,10^ In one study evaluating disparities across five health systems as part of a clinical trial, racial disparities disappeared when accounting for differences in discharge diagnoses such as opioid withdrawal or opioid overdose.^8^

Although racial and sex disparities in ED-initiated buprenorphine are clear, it is unknown how distinct patient presentations contribute to these disparities on a national level outside of a clinical trial setting. Using a nationally representative database, we aimed to quantify the contribution of diagnosis subtypes to the receipt of buprenorphine in the ED and determine whether racial and sex disparities in ED-initiated buprenorphine are robust to diagnosis outside of a clinical trial setting.

## METHODS

### Data source and study population

This is a cross-sectional analysis of opioid-related ED encounters from January 2020 through December 2023 using the Epic Cosmos database. The Epic Cosmos database is a research database maintained by Epic Systems and includes data from 1548 hospitals and 259 million patients. It is broadly representative of demographics of the United States. Encounters were included if they originated in the ED and had an opioid-related International Classification of Diseases 10 (ICD-10) billing diagnosis for the encounter. Billing codes representing opioid-related encounters were defined by the Healthcare Utilization Project.^11^ Encounters were excluded for incomplete data. Encounters were subcategorized by ICD-10 diagnosis code into subtypes of opioid withdrawal, opioid overdose, and other opioid complications (Appendix A).^12,13^ Subtypes were not mutually exclusive as individual encounters could belong to more than one subtype if multiple ICD-10 codes were entered.

### Statistical analysis

Using the sample of opioid-related ED encounters, we examined whether sex, race, or ethnicity differences in buprenorphine utilization existed when adjusting for encounter category subtype using a composite outcome of buprenorphine receipt, defined as administration or prescribing of buprenorphine. Buprenorphine was defined as any formulation of buprenorphine, including those that contain naloxone. We tested for interactions between demographic variables and encounter category to determine whether any such sex, race, or ethnicity disparities in buprenorphine administration were moderated by encounter category. Multivariable logistic regression models examined demographic differences in buprenorphine receipt and potential moderation by encounter subtype. Adjusted models included age, sex, race, ethnicity, rural– urban commuting area (RUCA), social vulnerability index (SVI), and encounter category. RUCA is a 1- to 10-point scale that delineates metropolitan, micropolitan, small town, and rural commuting areas based on a number of factors including size and population. Following recommended practices for interpretable categorizations, scores were aggregated to Categories 1–3, 4–6, and 7–10 representing urban, large rural city/town, and small and isolated small rural town, respectively, for analysis.^14^ SVI is a metric maintained by the Centers for Disease Control and Prevention that represents a composite measure of demographic and socioeconomic factors that affects communities with low scores representing areas with lowest social vulnerability.^15^ Adjusted odds ratios (ORs), 95% confidence intervals (CIs), and *p*-values were determined for all categories of covariates and potential confounders.

Following determination of the presence of interactions between demographic variables and subtypes of opioid-related encounters, subgroup regression models were built separately for these subtypes of opioid-related encounters representing opioid withdrawal and opioid overdose to evaluate for disparities in buprenorphine receipt within each subtype category based on sex, race, and ethnicity. Adjusted and unadjusted ORs were determined within encounter subtypes while controlling for demographic variables noted above as well as presence of other encounter category codes. While we controlled for demographic variables, we did perform a post hoc analysis to determine whether opioid diagnosis subtypes differ by demographic characteristics. Among variables used in modeling, a small amount of missing data existed only for RUCA (<0.01%) and SVI (1.68%), so a missing value category was included in regression analyses for both variables. As inclusion of a missing value category and treatment of SVI as a categorical variable based on quartiles yielded no discernible differences in results for any included model, results reported here include continuous SVI for ease of interpretation. This study adheres, where appropriate, to the STrengthening the Reporting of OBservational studies in Epidemiology (STROBE) guidelines (Appendix B) and was deemed exempt as non–human subjects research by the institutional review board of the primary institution.^16^

## RESULTS

We examined the available 1,088,033 opioid-related ED encounters included for analysis during the study period. Of these, 1,069,760 (98.3%) were used in logistic regression analyses, due to some missing SVI data. Demographic information for the study sample and subgroups is shown in Table 1. Male sex was associated with increased odds of buprenorphine administration or prescription (adjusted OR [aOR] 1.18, 95% CI 1.16–1.19) relative to female sex. All race categories except for American Indian/Alaska Native had lower odds of receiving buprenorphine compared to the reference category of White race (Table 2). Among categories of encounter type in adjusted models, the presence of opioid withdrawal codes was associated with more than double the odds of receiving buprenorphine (aOR 2.22, 95% CI 2.18–2.26), while overdose (aOR 0.52, 95% CI 0.51–0.53) and other complications (aOR 0.69, 95% CI 0.64–0.70) were associated with reduced odds of receiving buprenorphine.

Tests of encounter subtype by demographic variable interactions were all significant (*p* < 0.001), so emphasis was placed on subgroup analyses by encounter subtype for interpretability. The results of these analyses are shown in Table 3. The increased odds for buprenorphine receipt among male patients compared to female patients remained across both encounter category subtypes. Some racial disparities noted in the full sample were not noted in subtype-specific analyses. In the opioid withdrawal subtype, no statistically significant racial disparities existed apart from an increased odds of buprenorphine administration or prescription among American Indian/Native American (aOR 1.22, 95% CI 1.15–1.31) and Black (aOR 1.04, 95% CI 1.01–1.07) patients when compared with White patients. The latter difference contrasts with the decrease in odds of buprenorphine administration for Black patients among the full sample and opioid overdose category. Also, among the opioid overdose category, Hispanic patients had increased odds of receiving buprenorphine compared to non-Hispanic patients (aOR 1.08, 95% CI 1.02–1.14), a reversal of the trend observed in the withdrawal subgroup (aOR 0.90, 95% CI 0.86–0.93). A post hoc analysis of diagnosis code subtypes by sex, race, and ethnicity indicated that males were more likely to have overdose and withdrawal diagnosis codes than females and that patients representing racial minorities were more likely than White patients to have an overdose diagnosis code but less likely to have a withdrawal diagnosis code (Appendix C).

## DISCUSSION

**FIGURE 1:**
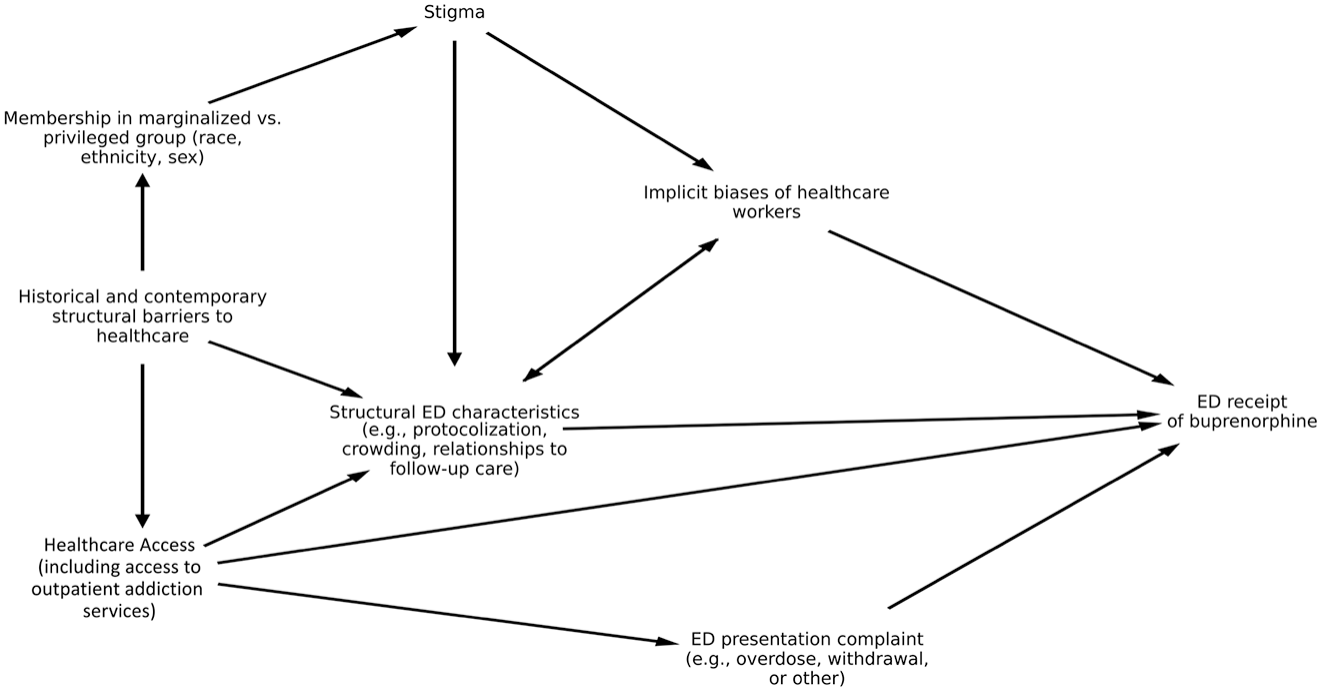
Directed acyclic graph of factors influencing the relationship between ED presenting complaint and receipt of buprenorphine.

Among this cohort of ED patients with an opioid-related ICD-10 code, the presence of opioid withdrawal was associated with over twice the odds of receiving buprenorphine. Racial and sex disparities were noted in the administration or prescription of buprenorphine. Male patients were more likely to receive buprenorphine compared to female patients, a finding that was robust to controlling for encounter subtype. Racial disparities were evident as well, with all race categories except American Indian/Alaska Native having lower odds of receiving buprenorphine relative to White patients. In subgroup analysis for those with opioid withdrawal, racial disparities among Black patients were reversed when compared to the full cohort and opioid overdose category. Disparities involving the receipt of buprenorphine in patients with opioid misuse in the ED are likely related to multiple factors including structural barriers to health care and stigma. We have conceptualized these factors in a directed acyclic graph (Figure 1) to identify potential confounders between presenting complaints and receipt of buprenorphine. We will address these factors here.^17^

The finding that patients with opioid withdrawal had the highest odds of receiving buprenorphine aligns with clinical expectations, as withdrawal is a well-established indication for buprenorphine treatment.^18^ This finding may reflect the need for more immediate treatment with buprenorphine during the acute opioid withdrawal period than with other opioid-related complications. Additionally, the increased use of buprenorphine likely reflects implementation of well-established protocols for the treatment of opioid withdrawal in the ED setting.^19-22^

The reduced odds of buprenorphine receipt among patients with overdose or other complications may indicate a lack of a protocolized approach for buprenorphine administration in many health systems. This may also be explained by the focus on acute stabilization rather than long-term treatment in the ED. In the emergent care of patients with acute opioid overdose, buprenorphine initiation should be considered only after stabilization. Clinicians may worry about a prolonged waiting period in the ED to avoid precipitating withdrawal with buprenorphine following stabilization although prehospital programs utilizing emergency medical services have successfully initiated buprenorphine in this context.^23^ The relatively short length of an ED encounter may encourage clinicians to refer for MOUD in the outpatient setting even with the knowledge that follow-up after referral from the ED is infrequently completed.^24,25^ In these cases, home induction is possible but requires system-level interventions such as proto-colization and appropriate follow-up care. ED clinicians may be hesitant to prescribe buprenorphine for home induction, fearing precipitated withdrawal or drug diversion.^26^ Further education and protocolization, including clinical decision support embedded within the electronic health record, should help to improve buprenorphine prescribing in this context.^22^

Subgroup analyses offer additional insights into disparities of buprenorphine administration or prescription. The consistently increased odds for receiving buprenorphine by male patients across all encounter subtypes highlights a pervasive and systemic disparity disadvantaging females with OUD seeking care in the ED.^27^ However, some racial disparities observed in the full sample were not present in specific encounter categories. This suggests that while racial disparities exist, the presence and nature of these disparities may differ based on the context of the encounter. The racial disparities noted in the full cohort may reflect a disparate use of buprenorphine for patients outside of the extremes for opioid-related complaints (i.e., withdrawal or overdose), intersectional stigma leading to disparate identification of opioid withdrawal by treating clinicians based on patient demographic variables, or different ED utilization patterns among racial subgroups. While disparities were noted in diagnosis subtype by race, we controlled for this in the regression models, indicating that disparities in buprenorphine receipt were not due to demographic variability in encounter subtype.

The observed sex disparities are likely multifactorial. In addition to implicit biases that may be held by individual clinicians, access to OUD treatment is frequently complicated by a range of factors that disproportionately affect female patients. The factors include an increased incidence of psychiatric disorders comorbid to substance misuse, a history of intimate partner violence and sexual abuse, and pregnancy-related stigmatization and criminalization associated with OUD.^27-31^ These experiences create barriers to healthcare treatment, engagement, and retention. Women who have experienced intimate partner violence or sexual abuse, which is more common among those with substance use disorders, may struggle with trust in medical systems, leading to hesitancy in seeking or adhering to treatment.^32-34^ Pregnant patients with a history of OUD may not seek out treatment for fear of encountering legal issues or facing social stigma. Comorbid psychiatric disorders, such as depression or anxiety, can exacerbate the challenges women face in engaging with and staying in treatment. The complexity of these factors necessitates a nuanced approach to OUD treatment, which may not be easily accomplished in ED settings without the presence of appropriate social support resources. Reducing sex-based disparities requires targeted interventions that recognize and address the unique challenges women face in accessing and maintaining OUD treatment.

Racial disparities in the opioid overdose group suggest systemic barriers in the initiation of buprenorphine treatment in the ED setting, particularly among non-White patients. Overdose deaths among Black patients surpassed that of White patients for the first time since 1999 in 2020, with a 16.3% higher overdose mortality rate for Black patients compared to White patients.^35^ American Indian or Alaska Native patients experienced the highest rate of mortality during this period and overdose rates among Hispanic patients also increased. These findings suggest that systemic factors may contribute to inequities in receiving OUD treatment. Home induction with outpatient follow-up, for example, can be difficult for patients with social barriers more prevalent among racial minorities such as limited access to health care resources, poor access to transportation, unstable housing, and insufficient support systems.^36^ Addressing these multifaceted barriers is essential for promoting equitable access to OUD treatment across races.

## LIMITATIONS

These results should be interpreted within the context of the study's limitations. As a retrospective study using data collected for nonresearch purposes, some data may not be accurate. Some variables, such as historic medications including previous buprenorphine prescriptions and other forms of MOUD, were unreliable in the Cosmos database and therefore not included for analysis. Other potentially important factors, such as outpatient referrals and the clinical opioid withdrawal scale, were similarly excluded due to inconsistent documentation from sites participating in Epic Cosmos. Additionally, while the database is broadly representative of the U.S. population, it may not perfectly represent all ED encounters. The SVI, while used as a marker for social vulnerability, is based on patient address and may not reflect individual circumstances. The use of ICD-10 codes in the identification of opioid-related encounters has its own limitations including low sensitivity, which likely led to an underdetection of opioid-related encounters.^37-39^ Additionally, a small amount of missingness existed in SVI that cannot be definitively established as missing at random, though reported results appear robust to inclusion of participants missing SVI data. Lastly, although we have focused on clinician and health system responsibilities for buprenorphine administration and prescribing, there are patient-level factors including hesitancy in taking MOUD.^26,40,41^

## CONCLUSIONS

Among patients with an opioid-related ED encounter, female patients and patients from most non-White racial groups are less likely to be administered or prescribed buprenorphine. Some racial disparities disappear when solely considering encounters for opioid withdrawal but persist among encounters involving opioid overdose. Further work must be done to determine the systemic causes underlying such disparities and expand protocols and resources to decrease disparities in buprenorphine receipt from the ED.

## Supplementary Material

Appendix C

Appendix A

Appendix B

## Figures and Tables

**TABLE 1:** Characteristics of patients with opioid-related ED encounters (*n* = 1,088,033).

	Full sample	Opioid withdrawal	Opioid overdose
Total	*n* = 1,088,033	*n* = 172,786	*n* = 179,843
Sex			
Female	462,455 (42.50)	71,450 (41.36)	66,723 (37.10)
Male	625,578 (57.50)	101,336 (58.65)	113,120 (62.90)
Age (years)	48.23 (±15.35)	43.97 (±14.22)	46.29 (±14.85)
Race			
American Indian or Alaska Native	25,852 (2.38)	4237 (2.45)	4671 (2.60)
Asian	7743 (0.71)	1337 (0.77)	1486 (0.83)
Black or African American	212,548 (19.54)	30.702 (17.77)	41,306 (22.97)
Native Hawaiian or Pacific Islander	2894 (0.27)	500 (0.29)	532 (0.30)
White	792,909 (72.88)	127,312 (73.68)	122,866 (68.32)
Other/missing	46,087 (4.24)	8698 (5.03)	8982 (5.99)
Ethnicity			
Hispanic	90,971 (8.36)	16,213 (9.38)	17,003 (9.45)
Non-Hispanic	997,062 (91.64)	156,573 (90.62)	162,840 (90.55)
Insurance			
Public (Medicare or Medicaid)	383,597 (35.26)	62,942 (36.43)	61,188 (34.02)
Private	616,620 (56.67)	91,814 (53.14)	100,263 (55.75)
Self-pay	57,446 (5.28)	12,689 (7.34)	12,757 (7.09)
SVI			
First quartile	194,737 (17.90)	31,571 (18.27)	29,809 (16.58)
Second quartile	227,916 (20.95)	36,026 (20.85)	36,498 (20.29)
Third quartile	276,596 (25.42)	43,178 (24.99)	45,498 (25.30)
Fourth quartile	370,497 (34.05)	58,778 (34.02)	64,745 (36.00)
Missing	18,287 (1.68)	3233 (1.87)	3293 (1.83)
RUCA code			
Urban	968,375 (89.00)	152,927 (88.51)	162,049 (90.11)
Large rural city/town	73,620 (6.77)	12,295 (7.12)	10,724 (5.96)
Small and isolated small rural town	45,941 (4.22)	7557 (4.37)	7049 (3.92)
Missing	97 (<0.01)	7 (<0.01)	21 (0.1)
Encounter categorization			
Opioid withdrawal	172,786 (15.88)	—	—
Opioid overdose	179,843 (16.53)	—	—
Other opioid complication	707,848 (65.06)	—	—

*Note*: Data are reported as *n* (%) or mean (±SD).

Abbreviations: RUCA, rural–urban commuting area; SVI, social vulnerability index.

**TABLE 2:** Associations between ED encounter characteristics and buprenorphine receipt.

Covariate	Unadjusted OR		Adjusted OR	
	(95% CI)	*p*-value	(95% CI)	*p*-value
Sex				
Female	1 [reference]		1 [reference]	
Male	1.18 (1.17–1.19)	<0.001	1.18 (1.16–1.19)	<0.001
Race				
White	1 [reference]		1 [reference]	
American Indian or Alaska Native	0.96 (0.93–0.99)	0.010	1.04 (1.00–1.08)	0.048
Asian	0.80 (0.75–0.85)	<0.001	0.85 (0.79–0.91)	<0.001
Black	0.72 (0.71–0.73)	<0.001	0.80 (0.79–0.81)	<0.001
Native Hawaiian or Pacific Islander	0.75 (0.68–0.83)	<0.001	0.79 (0.71–0.89)	<0.001
Other	0.87 (0.84–0.89)	<0.001	0.91 (0.75–0.94)	<0.001
Ethnicity				
Non-Hispanic	1 [reference]		1 [reference]	
Hispanic	0.98 (0.96–1.00)	0.012	0.98 (0.95–0.99)	0.034
RUCA				
Urban	1 [reference]		1 [REFERENCE]	
Large rural city/town	1.04 (1.03–1.07)	<0.001	0.90 (0.78–0.92)	<0.001
Small and isolated small rural town	1.12 (1.10–1.15)	<0.001	0.95 (0.92–0.98)	<0.001
Missing	2.03 (1.32–3.13)	0.001	2.42 (1.45–4.03)	0.001
Age	0.98 (0.98–0.98)	<0.001	0.99 (0.99–0.99)	0.99
SVI	1.00 (1.00–1.00)	<0.001	1.00 (1.00–1.00)	1.00
Opioid withdrawal	2.79 (2.76–2.82)	<0.001	2.22 (2.18–2.26)	<0.001
Opioid overdose	0.54 (0.53–0.55)	<0.001	0.52 (0.51–0.53)	<0.001
Other opioid complication	0.59 (0.59–0.59)	<0.001	0.69 (0.64–0.70)	<0.001

Abbreviations: RUCA, rural–urban commuting area; SVI, social vulnerability index.

**TABLE 3:** Buprenorphine receipt by encounter subtype.

	Opioid withdrawal		Opioid overdose	
Covariate	aOR (95% CI)	*p*-value	aOR (95% CI)	*p*-value
Sex				
Female	1 [reference]		1 [reference]	
Male	1.22 (1.20–1.25)	<0.001	1.12 (1.09–1.16)	<0.001
Race				
White	1 [reference]		1 [reference]	
American Indian or Alaska Native	1.22 (1.15–1.31)	<0.001	0.98 (0.89–1.08)	0.680
Asian	0.91 (0.81–1.03)	0.132	0.91 (0.77–1.07)	0.246
Black	1.04 (1.01–1.07)	0.010	0.84 (0.81–0.87)	<0.001
Native Hawaiian or Pacific Islander	0.88 (0.73–1.07)	0.192	0.92 (0.70–1.21)	0.564
Other	0.91 (0.86–0.97)	0.004	0.90 (0.82–0.99)	0.026
Ethnicity				
Non-Hispanic	1 [reference]		1 [reference]	
Hispanic	0.90 (0.86–0.93)	0.034	1.08 (1.02–1.14)	0.013
RUCA				
Urban	1 [reference]		1 [reference]	
Large rural city/town	0.77 (0.74–0.80)	<0.001	0.93 (0.87–0.99)	0.027
Small and isolated small rural town	0.82 (0.78–0.86)	<0.001	0.97 (0.90–1.05)	0.518
Missing	11.53 (1.34–99.60)	0.026	3.12 (1.18–8.22)	0.021
Age	0.99 (0.99–0.99)	<0.001	0.99 (0.99–0.99)	<0.001
SVI	1.00 (1.00–1.00)	<0.001	1.00 (1.00–1.00)	<0.001
Opioid withdrawal	—	—	4.45 (4.22–4.69)	<0.001
Opioid overdose	1.10 (1.05–1.16)	<0.001	—	—
Other opioid complication	0.89 (0.85–0.93)	<0.001	0.90 (0.87–=0.93)	<0.001

Abbreviations: aOR, adjusted odds ratio; RUCA, rural–urban commuting area; SVI, social vulnerability index.

## Data Availability

The data that support the findings of this study are available from Epic Cosmos. Restrictions apply to the availability of these data, which were used under license for this study. Data are available from https://cosmos.epic.com with the permission of Epic Cosmos.

## References

[1] Multiple Cause of Death 1999–2017 on CDC WONDER Online Database. Centers for Disease Control and Prevention, National Center for Health Statistics; 2020.

[2] Sordo L, Barrio G, Bravo MJ, Mortality risk during and after opioid substitution treatment: systematic review and meta-analysis of cohort studies. BMJ. 2017;357:j1550. 28446428 10.1136/bmj.j1550 PMC5421454

[3] Weiner SG, Little K, Yoo J, Opioid overdose after medication for opioid use disorder initiation following hospitalization or ED visit. JAMA Netw Open. 2024;7(7):e2423954. 39037812 10.1001/jamanetworkopen.2024.23954 PMC11265135

[4] Dowell D, Brown S, Gyawali S, Treatment for opioid use disorder: population estimates — United States, 2022. Morb Mortal Wkly Rep. 2024;73(25):567–574. 10.15585/mmwr.mm7325a1 PMC11254342 38935567

[5] Beckerleg W, Hudgins J. Substance use-related emergency department visits and resource utilization. West J Emerg Med. 2022;23(2): 166–173. 35302449 10.5811/westjem.2022.1.53834 PMC8967472

[6] Kanzaria HK, Niedzwiecki M, Cawley CL, Frequent emergency department users: focusing solely on medical utilization misses the whole person. Health Aff (Millwood). 2019;38(11):1866–1875. 31682499 10.1377/hlthaff.2019.00082

[7] D'Onofrio G, McCormack RP, Hawk K. Emergency departments – a 24/7/365 option for combating the opioid crisis. N Engl J Med. 2018;379(26):2487–2490. 30586522 10.1056/NEJMp1811988

[8] Holland WC, Li F, Nath B, Racial and ethnic disparities in emergency department-initiated buprenorphine across five health care systems. Acad Emerg Med. 2023;30(7):709–720. 36660800 10.1111/acem.14668 PMC10467357

[9] Papp J, Emerman C. Disparities in emergency department naloxone and buprenorphine initiation. West J Emerg Med. 2023;24(4): 710–716. 37527392 10.5811/westjem.58636 PMC10393464

[10] Chhabra N, Smith D, Dickinson G, Trends and disparities in initiation of buprenorphine in US emergency departments, 2013-2022. JAMA Netw Open. 2024;7(9):e2435603. 39325455 10.1001/jamanetworkopen.2024.35603 PMC11428009

[11] Weiss AJ, McDermott KW, Heslin KC. Opioid-related hospital stays among women in the United States, 2016: statistical brief #247. Healthcare Cost and Utilization Project (HCUP) Statistical Briefs. Agency for Healthcare Research and Quality (US); 2006. https://www.ncbi.nlm.nih.gov/books/NBK538344/30855771

[12] Slavova S, Quesinberry D, Costich JF, ICD-10-CM-based definitions for emergency department opioid poisoning surveillance: electronic health record case confirmation study. Public Health Rep. 2020;135(2):262–269. 32040923 10.1177/0033354920904087 PMC7036606

[13] Glanz JM, Binswanger IA, Shetterly SM, Narwaney KJ, Xu S. Association between opioid dose variability and opioid overdose among adults prescribed long-term opioid therapy. JAMA Netw Open. 2019;2(4):e192613. 31002325 10.1001/jamanetworkopen.2019.2613 PMC6481879

[14] RUCA Data. Rural Health Research Center. 2024. Accessed October 10, 2024. https://depts.washington.edu/uwruca/ruca-uses.php

[15] Social Vulnerability Index. Agency for Toxic Substances and Disease Registry, Centers for Disease Control and Prevention. 2023. Accessed October 10, 2024. https://www.atsdr.cdc.gov/place-health/php/svi/?CDC_AAref_Val=https://www.atsdr.cdc.gov/placeandhealth/svi/index.html

[16] von Elm E, Altman DG, Egger M, The Strengthening the Reporting of Observational Studies in Epidemiology (STROBE) statement: guidelines for reporting observational studies. Lancet Lond Engl. 2007;370(9596):1453–1457. 10.1016/S0140-6736(07)61602-X 18064739

[17] Howe CJ, Bailey ZD, Raifman JR, Jackson JW. Recommendations for using causal diagrams to study racial health disparities. Am J Epidemiol. 2022;191(12):1981–1989. 35916384 10.1093/aje/kwac140 PMC10144617

[18] Gowing L, Ali R, White JM, Mbewe D. Buprenorphine for managing opioid withdrawal. Cochrane Database Syst Rev. 2017;2(2):CD002025. doi:10.1002/14651858.CD002025.pub5 28220474 PMC6464315

[19] Torres-Lockhart KE, Lu TY, Weimer MB, Stein MR, Cunningham CO. Clinical management of opioid withdrawal. Addiction. 2022;117(9):2540–2550. 35112746 10.1111/add.15818

[20] D'Onofrio G, Edelman EJ, Hawk KF, Implementation facilitation to promote emergency department-initiated buprenorphine for opioid use disorder. JAMA Netw Open. 2023;6(4):e235439. doi:10.1001/jamanetworkopen.2023.5439 37017967 PMC10077107

[21] Lowenstein M, Perrone J, McFadden R, Impact of universal screening and automated clinical decision support for the treatment of opioid use disorder in emergency departments: a difference-indifferences analysis. Ann Emerg Med. 2023;82(2):131–144. 37318434 10.1016/j.annemergmed.2023.03.033 PMC11019868

[22] Melnick ER, Jeffery MM, Dziura JD, User-centred clinical decision support to implement emergency department-initiated buprenorphine for opioid use disorder: protocol for the pragmatic group randomised EMBED trial. BMJ Open. 2019;9(5):e028488. 10.1136/bmjopen-2018-028488 PMC6550013 31152039

[23] Carroll G, Solomon KT, Heil J, Impact of administering buprenorphine to overdose survivors using emergency medical services. Ann Emerg Med. 2023;81(2):165–175. 36192278 10.1016/j.annemergmed.2022.07.006

[24] Jaeger S, Fuehrlein B. Buprenorphine initiation to treat opioid use disorder in emergency rooms. J Neurol Sci. 2020;411:116716. 32097813 10.1016/j.jns.2020.116716

[25] D'Onofrio G, O'Connor PG, Pantalon MV, Emergency department-initiated buprenorphine/naloxone treatment for opioid dependence: a randomized clinical trial. JAMA. 2015;313(16):1636–1644. doi:10.1001/jama.2015.3474 25919527 PMC4527523

[26] Hawk KF, D'Onofrio G, Chawarski MC, Barriers and facilitators to clinician readiness to provide emergency department-initiated buprenorphine. JAMA Netw Open. 2020;3(5):e204561. 32391893 10.1001/jamanetworkopen.2020.4561 PMC7215257

[27] Goetz TG, Becker JB, Mazure CM. Women, opioid use and addiction. FASEB J. 2021;35(2):e21303. 33433026 10.1096/fj.202002125R

[28] Weber A, Miskle B, Lynch A, Arndt S, Acion L. Substance use in pregnancy: identifying stigma and improving care. Subst Abus Rehabil. 2021;12:105–121. doi:10.2147/SAR.S319180 PMC8627324 34849047

[29] Greenfield SF, Brooks AJ, Gordon SM, Substance abuse treatment entry, retention, and outcome in women: a review of the literature. Drug Alcohol Depend. 2007;86(1):1–21. 16759822 10.1016/j.drugalcdep.2006.05.012 PMC3532875

[30] Barbosa-Leiker C, McPherson S, Layton ME, Burduli E, Roll JM, Ling W. Sex differences in opioid use and medical issues during buprenorphine/naloxone treatment. Am J Drug Alcohol Abuse. 2018;44(4):488–496. 29672167 10.1080/00952990.2018.1458234 PMC6186400

[31] Zilberman ML, Tavares H, Blume SB, el-Guebaly N. Substance use disorders: sex differences and psychiatric comorbidities. Can J Psychiatr. 2003;48(1):5–13. doi:10.1177/070674370304800103 12635558

[32] Armstrong EM, Glover Reed B, Bennett LW. How and how much: combined services for domestic violence and substance abuse. Violence Women. 2019;25(12):1450–1470. 10.1177/1077801218820201 30600781

[33] McLean K, Murphy J, Kruis N. “I think we're getting better but we're still not there”: provider-based stigma and perceived barriers to care for people who use opioids (PWUO). J Subst Use Addict Treat. 2024;159:209270. 38103831 10.1016/j.josat.2023.209270

[34] Najavits LM, Sonn J, Walsh M, Weiss RD. Domestic violence in women with PTSD and substance abuse. Addict Behav. 2004;29(4):707–715. 15135552 10.1016/j.addbeh.2004.01.003

[35] Friedman JR, Hansen H. Evaluation of increases in drug overdose mortality rates in the US by race and ethnicity before and during the COVID-19 pandemic. JAMA Psychiatry. 2022;79(4):379–381. doi:10.1001/jamapsychiatry.2022.0004 35234815 PMC8892360

[36] Brandt EJ. Social determinants of racial health inequities. Lancet Public Health. 2023;8(6):e396–e397. 37244668 10.1016/S2468-2667(23)00100-7 PMC11104490

[37] Chhabra N, Smith D, Pachwicewicz P, Performance of international classification of Disease-10 codes in detecting emergency department patients with opioid misuse. Addiction. 2024;119(4):766–771. doi:10.1111/add.16394 38011858 PMC11162597

[38] Rowe CL, Santos G-M, Kornbluh W, Bhardwaj S, Faul M, Coffin PO. Using ICD-10-CM codes to detect illicit substance use: a comparison with retrospective self-report. Drug Alcohol Depend. 2021;221:108537. 33621806 10.1016/j.drugalcdep.2021.108537 PMC11008535

[39] Marks LR, Nolan NS, Jiang L, Muthulingam D, Liang SY, Durkin MJ. Use of ICD-10 codes for identification of injection drug use-associated infective endocarditis is nonspecific and obscures critical findings on impact of medications for opioid use disorder. Open Forum Infect Dis. 2020;7(10):ofaa414. 33094117 10.1093/ofid/ofaa414 PMC7566393

[40] Calcaterra SL, Martin M, Englander H. Identifying barriers to OUD treatment linkage from the emergency department to the community. JAMA Netw Open. 2023;6(5):e2312683. 37163270 10.1001/jamanetworkopen.2023.12683 PMC10896496

[41] Kelly TD, Hawk KF, Samuels EA, Strayer RJ, Hoppe JA. Improving uptake of emergency department-initiated buprenorphine: barriers and solutions. West J Emerg Med. 2022;23(4):461–467. 35980414 10.5811/westjem.2022.2.52978 PMC9391022

