# Supplementary material for: Racial, ethnic, and sex disparities in buprenorphine treatment from emergency departments by discharge diagnosis
Source: Acad Emerg Med. 2025 Apr 25;32(9):985–93. doi: 10.1111/acem.70035 (PMC12353228; doi:10.1111/acem.70035)
Supplement: Supplementary file 1 — Appendix A. [file ACEM-32-985-s001.docx]

Appendix A

(Derived from HCUP^2^)

Withdrawal

| F11.23 | Opioid dependence with withdrawal |
| --- | --- |
| F11.93 | Opioid use, unspecified, with withdrawal |

Overdose/Intoxication^3,4^

| F11.120 | Opioid abuse with intoxication, uncomplicated | |
| --- | --- | --- |
| F11.121 | Opioid abuse with intoxication, delirium | |
| F11.122 | Opioid abuse with intoxication, with perceptual disturbance | |
| F11.129 | Opioid abuse with intoxication, unspecified | |
| F11.220 | Opioid dependence with intoxication, uncomplicated | |
| F11.221 | Opioid dependence with intoxication, delirium | |
| F11.222 | Opioid dependence with intoxication, with perceptual disturbance | |
| F11.229 | Opioid dependence with intoxication, unspecified | |
| F11.90 | Opioid use, unspecified, uncomplicated | |
| F11.920 | Opioid use, unspecified with intoxication, uncomplicated | |
| F11.921 | Opioid use, unspecified with intoxication delirium | |
| F11.922 | Opioid use, unspecified with intoxication, with perceptual disturbance | |
| F11.929 | Opioid use, unspecified with intoxication, unspecified | |
| T40.0X1A | Poisoning by opium, accidental (unintentional), initial encounter | |
| T40.0X1D | Poisoning by opium, accidental (unintentional), subsequent encounter | |
| T40.0X2A | Poisoning by opium, intentional self-harm, initial encounter | |
| T40.0X2D | Poisoning by opium, intentional self-harm, subsequent encounter | |
| T40.0X3A | Poisoning by opium, assault, initial encounter | |
| T40.0X3D | Poisoning by opium, assault, subsequent encounter | |
| T40.0X4A | Poisoning by opium, undetermined, initial encounter | |
| T40.0X4D | Poisoning by opium, undetermined, subsequent encounter | |
| T40.1X1A | Poisoning by heroin, accidental (unintentional), initial encounter | |
| T40.IXID | Poisoning by heroin, accidental (unintentional), subsequent encounter | |
| T40.1X2A | Poisoning by heroin, intentional self-harm, initial encounter | |
| T40.1X2D | Poisoning by heroin, intentional self-harm, subsequent encounter | |
| T40.1X3A | Poisoning by heroin, assault, initial encounter | |
| T40.1X3D | Poisoning by heroin, assault, subsequent encounter | |
| T40.1X4A | Poisoning by heroin, undetermined, initial encounter | |
| T40.1X4D | Poisoning by heroin, undetermined, subsequent encounter | |
| T40.2X1A | Poisoning by other opioids, accidental (unintentional), initial encounter | |
| T40.2X1D | Poisoning by other opioids, accidental (unintentional), subsequent encounter | |
| T40.2X2A | Poisoning by other opioids, intentional self-harm, initial encounter | |
| T40.2X2D | Poisoning by other opioids, intentional self-harm, subsequent encounter | |
| T40.2X3A | Poisoning by other opioids, assault, initial encounter | |
| T40.2X3D | Poisoning by other opioids, assault, subsequent encounter | |
| T40.2X4A | Poisoning by other opioids, undetermined, initial encounter | |
| T40.2X4D | Poisoning by other opioids, undetermined, subsequent encounter | |
| T40.3X1A | | Poisoning by methadone, accidental (unintentional), initial encounter |
| T40.3X1D | | Poisoning by methadone, accidental (unintentional), subsequent encounter |
| T40.3X2A | | Poisoning by methadone, intentional self-harm, initial encounter |
| T40.3X2D | | Poisoning by methadone, intentional self-harm, subsequent encounter |
| T40.3X3A | | Poisoning by methadone, assault, initial encounter |
| T40.3X3D | | Poisoning by methadone, assault, subsequent encounter |
| T40.3X4A | | Poisoning by methadone, undetermined, initial encounter |
| T40.3X4D | | Poisoning by methadone, undetermined, subsequent encounter |
| T40.4X1A | | Poisoning by synthetic narcotics, accidental (unintentional), initial encounter |
| T40.4X1D | | Poisoning by synthetic narcotics, accidental (unintentional), subsequent encounter |
| T40.4X2A | | Poisoning by other synthetic narcotics, intentional self-harm, initial encounter |
| T40.4X2D | | Poisoning by other synthetic narcotics, intentional self-harm, subsequent encounter |
| T40.4X3A | | Poisoning by other synthetic narcotics, assault, initial encounter |
| T40.4X3D | | Poisoning by other synthetic narcotics, assault, subsequent encounter |
| T40.4X4A | | Poisoning by synthetic narcotics, undetermined, initial encounter |
| T40.4X4D | | Poisoning by synthetic narcotics, undetermined, subsequent encounter |
| T40.601A | | Poisoning by unspecified narcotics, accidental (unintentional), initial encounter |
| T40.601D | | Poisoning by unspecified narcotics, accidental (unintentional), subsequent encounter |
| T40.602A | | Poisoning by unspecified narcotics, intentional self-harm, initial encounter |
| T40.602D | | Poisoning by unspecified narcotics, intentional self-harm, subsequent encounter |
| T40.603A | | Poisoning by unspecified narcotics, assault, initial encounter |
| T40.603D | | Poisoning by unspecified narcotics, assault, subsequent encounter |
| T40.604A | | Poisoning by unspecified narcotics, undetermined, initial encounter |
| T40.604D | | Poisoning by unspecified narcotics, undetermined, subsequent encounter |
| T40.691A | | Poisoning by other narcotics, accidental (unintentional), initial encounter |
| T40.691D | | Poisoning by other narcotics, accidental (unintentional), subsequent encounter |
| T40.692A | | Poisoning by other narcotics, intentional self-harm, initial encounter |
| T40.692D | | Poisoning by other narcotics, intentional self-harm, subsequent encounter |
| T40.693A | | Poisoning by other narcotics, assault, initial encounter |
| T40.693D | | Poisoning by other narcotics, assault, subsequent encounter |
| T40.694A | | Poisoning by other narcotics, undetermined, initial encounter |
| T40.694D | | Poisoning by other narcotics, undetermined, subsequent encounter |

Other

| **ICD-10-CM diagnosis codes defining opioid-related inpatient stays** | |
| --- | --- |
| **ICD-10-CM diagnosis code** | **Description** |
| F11.10 | Opioid abuse, uncomplicated |
| F11.14 | Opioid abuse with opioid-induced mood disorder |
| F11.150 | Opioid abuse with opioid-induced psychotic disorder, with delusions |
| F11.151 | Opioid abuse with opioid-induced psychotic disorder, with hallucinations |
| F11.159 | Opioid abuse with opioid-induced psychotic disorder, unspecified |
| F11.181 | Opioid abuse with opioid-induced sexual dysfunction |
| F11.182 | Opioid abuse with opioid-induced sleep disorder |
| F11.188 | Opioid abuse with other opioid-induced disorder |
| F11.19 | Opioid abuse with unspecified opioid-induced disorder |
| F11.20 | Opioid dependence, uncomplicated |
| F11.24 | Opioid dependence with opioid-induced mood disorder |
| F11.250 | Opioid dependence with opioid-induced psychotic disorder, with delusions |
| F11.251 | Opioid dependence with opioid-induced psychotic disorder, with hallucinations |
| F11.259 | Opioid dependence with opioid-induced psychotic disorder, unspecified |
| F11.281 | Opioid dependence with opioid-induced sexual dysfunction |
| F11.282 | Opioid dependence with opioid-induced sleep disorder |
| F11.288 | Opioid dependence with other opioid-induced disorder |
| F11.29 | Opioid dependence with unspecified opioid-induced disorder |
| F11.94 | Opioid use, unspecified, with opioid-induced mood disorder |
| F11.950 | Opioid use, unspecified with opioid-induced psychotic disorder, with delusions |
| F11.951 | Opioid use, unspecified with opioid-induced psychotic disorder, with hallucinations |
| F11.959 | Opioid use, unspecified with opioid-induced psychotic disorder, unspecified |
| F11.981 | Opioid use, unspecified with opioid-induced sexual dysfunction |
| F11.982 | Opioid use, unspecified with opioid-induced sleep disorder |
| F11.988 | Opioid use, unspecified with other opioid-induced disorder |
| F11.99 | Opioid use, unspecified, with unspecified opioid-induced disorder |
| T40.0X5A | Adverse effect of opium, initial encounter |
| T40.0X5D | Adverse effect of opium, subsequent encounter |
| T40.2X5A | Adverse effect of other opioids, initial encounter |
| T40.2X5D | Adverse effect of other opioids, subsequent encounter |
| T40.3X5A | Adverse effect of methadone, initial encounter |
| T40.3X5D | Adverse effect of methadone, subsequent encounter |
| T40.4X5A | Adverse effect of synthetic narcotics, initial encounter |
| T40.4X5D | Adverse effect of synthetic narcotic, subsequent encounter |
| T40.605A | Adverse effect of unspecified narcotics, initial encounter |
| T40.605D | Adverse effect of unspecified narcotics, subsequent encounter |
| T40.695A | Adverse effect of other narcotics, initial encounter |
| T40.695D | Adverse effect of other narcotics, subsequent encounter |
| Abbreviation: ICD-10-CM, International Classification of Diseases, Tenth Revision, Clinical Modification | |

References

1. Holland WC, Li F, Nath B, et al. Racial and ethnic disparities in emergency department-initiated buprenorphine across five health care systems. *Acad Emerg Med Off J Soc Acad Emerg Med*. Published online January 19, 2023. doi:10.1111/acem.14668

2. Hospital Burden of Opioid-Related Inpatient Stays: Metropolitan and Rural Hospitals, 2016 #258. Accessed May 21, 2024. https://hcup-us.ahrq.gov/reports/statbriefs/sb258-Opioid-Hospitalizations-Rural-Metro-Hospitals-2016.jsp

3. Slavova S, Quesinberry D, Costich JF, et al. ICD-10-CM-Based Definitions for Emergency Department Opioid Poisoning Surveillance: Electronic Health Record Case Confirmation Study. *Public Health Rep Wash DC 1974*. 2020;135(2):262-269. doi:10.1177/0033354920904087

4. Glanz JM, Binswanger IA, Shetterly SM, Narwaney KJ, Xu S. Association Between Opioid Dose Variability and Opioid Overdose Among Adults Prescribed Long-term Opioid Therapy. *JAMA Netw Open*. 2019;2(4):e192613. doi:10.1001/jamanetworkopen.2019.2613
