# Supplementary material for: Racial, ethnic, and sex disparities in buprenorphine treatment from emergency departments by discharge diagnosis
Source: Acad Emerg Med. 2025 Apr 25;32(9):985–93. doi: 10.1111/acem.70035 (PMC12353228; doi:10.1111/acem.70035)
Supplement: Supplementary file 3 — Appendix C. [file ACEM-32-985-s003.docx]

Appendix C. Demographic differences in opioid diagnosis types

|  | Opioid withdrawal |  | Opioid overdose |  | Other diagnosis |  |
| --- | --- | --- | --- | --- | --- | --- |
| Covariate | aOR (95% CI) | p | aOR (95% CI) | p | aOR (95% CI) | p |
| Sex |  |  |  |  |  |  |
| Female | [Reference] |  | [Reference] |  | [Reference] |  |
| Male | 1.22 (1.02, 1.03) | <.001 | 1.28 (1.26, 1.29) | < .001 | 0.90 (0.89, 0.91) | <.001 |
| Race |  |  |  |  |  |  |
| White | [Reference] |  | [Reference] | < .001 | [Reference] |  |
| AI/NA | 0.97 (0.94, 1.01) | .067 | 1.15 (1.11, 1.18) | < .001 | 0.97 (0.95, 1.00) | .056 |
| Asian | 0.97 (0.92, 1.03) | .395 | 1.20 (1.13, 1.27) | < .001 | 0.96 (0.92, 1.01) | .113 |
| Black | 0.91 (0.89, 0.92) | < .001 | 1.27 (1.26, 1.29) | < .001 | 1.01 (1.00, 1.02) | .204 |
| NH/PI | 1.00 (0.91, 1.10) | .979 | 1.15 (1.04, 1.26) | .005 | 1.00 (0.92, 1.08) | .963 |
| Other | 1.08 (1.04, 1.11) | < .001 | 1.06 (1.03, 1.10) | < .001 | 0.97 (0.95, 1.00) | .033 |
| Ethnicity |  |  |  |  |  |  |
| Non-Hispanic | [Reference] |  | [Reference] |  | [Reference] |  |
| Hispanic | 0.96 (0.94, 0.99) | .001 | 0.94 (0.92, 0.96) | < .001 | 1.07 (1.05, 1.09) | < .001 |
| RUCA |  |  |  |  |  |  |
| Urban | [Reference] |  | [Reference] |  | [Reference] |  |
| Large rural city/town | 1.04 (1.02, 1.06) | < .001 | 0.87 (0.86, 0.89) | < .001 | 0.97 (0.95, 0.98) | < .001 |
| Small and isolated small rural town | 1.02 (0.99, 1.04) | .204 | 0.93 (0.91, 0.95) | < .001 | 0.91 (0.89, 0.93) | < .001 |
| Missing | 0.44 (0.19, 1.01) | .059 | 1.58 (0.97, 2.57) | .066 | 1.00 (0.65, 1.54) | .995 |
| Age | 0.98 (0.98, 0.98) | < .001 | 0.99 (0.99, 0.99) | < .001 | 1.02 (1.02, 1.02) | <.001 |
| Social Vulnerability Index | 1.00 (1.00, 1.00) | .642 | 1.00 (1.00, 1.00) | < .001 | 1.00 (1.00, 1.00) | .383 |
